# ITGB1 as a prognostic biomarker correlated with immune suppression in gastric cancer

**DOI:** 10.1002/cam4.5042

**Published:** 2022-07-21

**Authors:** Wenchao Gu, Hui Sun, Meng Zhang, Shaocong Mo, Cong Tan, Shujuan Ni, Zongcheng Yang, Yulin Wang, Weiqi Sheng, Lei Wang

**Affiliations:** ^1^ Department of Radiology Fudan University Shanghai Cancer Center Shanghai China; ^2^ Department of Diagnostic and Interventional Radiology University of Tsukuba Ibaraki Japan; ^3^ Department of Diagnostic Radiology and Nuclear Medicine Gunma University Graduate School of Medicine Maebashi Japan; ^4^ Department of Pathology Fudan University Shanghai Cancer Center Shanghai China; ^5^ Department of Oncology Shanghai Medical College of Fudan University Shanghai China; ^6^ Department of digestive diseases, Huashan Hospital Fudan University Shanghai China; ^7^ Center of stomatology, The First Affiliated Hospital of USTC, Division of Life Sciences and Medicine University of Science and Technology of China Hefei China; ^8^ Department of Nephrology, Zhongshan Hospital Fudan University Shanghai China

**Keywords:** gastric cancer, immunotherapy, ITGB1, tumor immune suppression, Wnt/β‐catenin signaling pathway

## Abstract

**Introduction:**

Gastric cancer is one of the common malignant tumors with a high incidence and mortality in China. Prognostic biomarkers and potential predictors of the treatment efficacy of gastric cancer urgently need to be identified. Integrin‐β (ITGB) is a superfamily of integrins and is involved in cell adhesion, tissue repair, immune response, and tumor metastasis.

**Methods:**

We analyzed ITGB1 expression in our hospital samples of the gastric cancer cohort. And the public data of The Cancer Genome Atlas stomach adenocarcinoma (TCGA‐STAD), The Asian Cancer Research Group (ACRG)/GSE62254, and GSE15459 data sets were analyzed by using the bioinformatic methods. The relationships between ITGB1 expression and clinicopathological features, patient prognosis, activation of the Wnt/β‐catenin signaling pathway, and tumor immunosuppressive factors were also explored.

**Results:**

The positive rate of ITGB1 expression in the Fudan University Shanghai Cancer Center gastric cancer tumor tissues was 61.4% (258/420) and correlated with deep invasion (*p* = 0.017), an advanced clinical stage (*p* = 0.011), and a poor prognosis (*p* < 0.05). The TCGA‐STAD/ACRG/GSE15459 cohorts also showed similar results. ITGB1 is one of the upstream molecules of the Wnt/β‐catenin signaling pathway and is correlated with tumor immune suppression. In gastric cancer, we found a correlation between ITGB1 expression and Wnt/β‐catenin signaling pathway activity. In the TCGA‐STAD/ACRG/GSE15459 cohorts, ITGB1 expression was positively associated with immunosuppressive factors and negatively associated with immunoactive factors. Patients with low ITGB1 expression exhibited a significantly high immunotherapy response ratio according to an analysis of tumor immune dysfunction and exclusion (TIDE), which may indicate that ITGB1 is a potential predictor of immunotherapy efficacy.

**Conclusions:**

ITGB1 affects the prognosis in gastric cancer patients and plays a core role in immune suppression in gastric cancer.

## Graphical abstract

The pancancer analysis of ITGB1

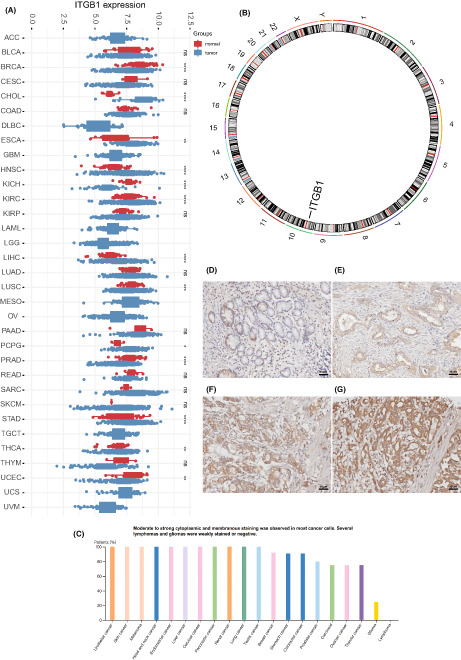

## 1 INTRODUCTION

Gastric cancer (GC) has become the second most common cause of cancer death in China.^1^ Immunotherapy has been a landmark advancement in the treatment of cancers over the last decade, including melanoma, lung cancer, and hepatocellular carcinoma.^2^, ^3^, ^4^, ^5^ However, not all GC patients can benefit from immunotherapy. Thus, identifying reliable prognostic predictors and immune escape‐related molecules in GC is important.

Integrins are transmembrane adhesion receptors consisting of alpha and beta subunits. Integrins involve cell–cell and cell–matrix interactions by interacting with the extracellular matrix, including cadherins, immunoglobulin, selectins, and syndecans. Integrin‐β (ITGB) is a superfamily of integrins and plays crucial roles in cell adhesion, proliferation, and differentiation.^6^ Upregulated ITGB1 resulted in cancer cell invasion and was correlated with a poor prognosis in GC.^7^, ^8^ β‐catenin is one of the core structural components of the cadherin–protein complex, which is critical for the activation of the Wnt/β‐catenin signaling pathway. ITGB1 has been shown to stimulate Wnt/β‐catenin signaling in lung cancer by inducing β‐catenin expression.^9^ Lv et al. found that collagen/ITGB1 promoted GC progression through the BCL9L/β‐catenin signaling pathway.^10^ Activation of this pathway occurs in numerous malignancies and correlates with tumor immune suppression. Aberrant integrin and other cell adhesion molecule expression also play a crucial role in tumor immunology.^11^

In GC, the underlying mechanisms of tumorigenesis induced by ITGB1 remain poorly understood. A few published studies mostly focused on ITGB1/FAK signaling^12^, ^13^ and the hTERT/ITGB1 pathway involved in the progression of GC.^14^, ^15^ However, the relationship between ITGB1 and the tumor microenvironment has not been evaluated. In this study, we comprehensively explored ITGB1 expression in GC and its correlations with clinicopathological features, prognosis, the Wnt/β‐catenin signaling pathway, and tumor immune suppression and predicted the possibility of ITGB1 as a potentially important target for immunotherapy in our own cohort and public data sets.

## 2 MATERIALS AND METHODS

### 2.1 Tumor specimens

This study was approved by the Clinical Research Ethics Committee of Fudan University Shanghai Cancer Center (FUSCC). Four hundred and twenty primary gastric carcinoma cases were retrieved from the files of the Department of Pathology, Fudan University Shanghai Cancer Center. Informed consent was acquired from all patients. All these patients in this study underwent radical gastrectomy without preoperative chemotherapy from 2010 to 2012. Two pathologists (Lei Wang and Meng Zhang) reviewed all the cases and confirmed the pathological diagnoses without discrepancies. Clinical information, including sex, age, tumor location, tumor size, therapeutic strategy, and clinical outcomes, was collected from medical records and pathology reports.

### 2.2 Immunohistochemical staining

Four‐micrometer‐thick sections of formalin‐fixed, paraffin‐embedded tissue were used for immunohistochemical staining. Immunostaining was performed using a Benchmark Ultra automated stainer (Ventana Medical Systems) according to the instructions. The primary antibodies included anti‐ITGB1 monoclonal antibody (Cat. No. ab52971, Abcam), anti‐β‐catenin monoclonal antibody (Cat. No. 610154, BD Biosciences), and anti‐IL‐10 monoclonal antibody (Cat. No mab91842, R&D Systems). Isotype‐specific immunoglobulins were used as negative controls by omitting the primary antibody.

### 2.3 Immunohistochemical evaluation

Immunohistochemistry (IHC) sections were reviewed by two pathologists (Lei Wang and Meng Zhang) before access the clinical information. For the ITGB1 IHC staining of each case, the results were scored using the H‐score, which was calculated by multiplying the percentage of positive tumor cells with the staining intensity (0, negative; 1, weak; 2, moderate; 3 strong). The final H‐score, the average of the duplicate scores from the two pathologists, was in the range of 0–300.^16^ A tumor with a final score ≥90 was considered to be an ITGB1‐positive expression case. β‐catenin staining was separated into four patterns: (1) loss of expression, (2) membranous expression, (3) cytoplasmic overexpression, and (4) nuclear accumulation. As for the nuclear expression pattern, β‐catenin nuclear staining was detected in more than 10% of the tumor cells.

### 2.4 Data acquisition

The TCGA database was used to collect TCGA‐STAD level 3 gene expression count data and somatic mutation data (http://portal.gdc.cancer.gov/). The expression data (count data) were converted into TPM (transcripts per kilobase million) values for further investigation. From the Gene Expression Omnibus (GEO; https://www.ncbi.nlm.nih.gov/geo/), the ACRG/GSE62254 and GSE15459 data sets were obtained. Background correction and quantile normalization for raw data from “CEL” files were done with the “affy” package. Finally, 864 patients from the TCGA‐STAD (*n* = 372), ACRG cohorts (*n* = 300), and GSE15459 (*n* = 192) were enrolled in this study for further analysis. The 33 independent TCGA cancer cohorts were enrolled in pan‐caner analysis, which contained 9703 tumor samples were retrieved through the UCSC Xena browser (http://xena.ucsc.edu/). The Human Protein Atlas was used to assess protein expression levels in pan‐cancer data (https://www.proteinatlas.org/).

### 2.5 Functional analysis and gene set enrichment analysis

The gene set (c2.cp.kegg.v7.2) enrichment analysis (GSEA) was performed using the R package “ClusterProfiler”.^17^ Single‐sample gene set enrichment analysis (ssGSEA) with tumor microenvironment (TME) signatures and immune cell signatures was performed as previously described.^18^

### 2.6 Survival prognosis analysis

To determine which GC patients belonged to the high‐ and low‐ITGB1 expression subgroups, we performed Kaplan–Meier analysis with the “survminer” R package. Further, to determine the significance of differences, the log‐rank test was performed. The FUSCC and ARCG cohorts were studied in terms of overall survival (OS) and disease‐free survival (DFS). The TCGA‐STAD and GES15459 cohorts were evaluated only by OS analysis.

### 2.7 Tumor mutation burden and prediction of immunotherapy response

The landscape of mutations in each patient based on ITGB1 expression in the TCGA‐STAD cohort was calculated by the “maftool” package.^19^ The total number of nonsynonymous mutations per megabase was used to calculate the tumor mutation burden (TMB).^20^ As previously described, the response to immune checkpoint blockade was predicted by Tumor Immune Dysfunction and Exclusion (TIDE) method.^21^, ^22^

### 2.8 Statistical analysis

R software (version: 4.03) was used to conduct all statistical analyses. The correlation coefficient was calculated by the Pearson analysis. The parametric test (Student's *t* test) or nonparametric test (Wilcoxon rank‐sum test) was performed to compare two groups. The *p* < 0.05 was considered the statistically significant difference.

## 3 RESULTS

### 3.1 ITGB1 overexpressed in pan‐cancer

**FIGURE 1:**
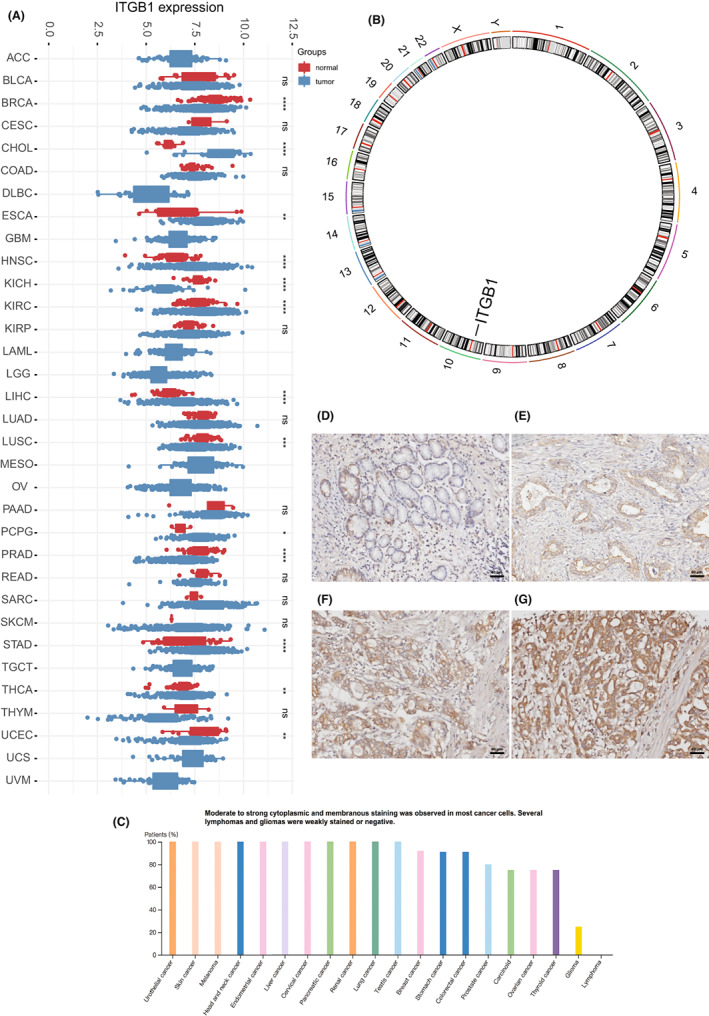
ITGB1 expression in normal and tumor tissue. (A) Pan‐cancer analysis of ITGB1 expression in normal tissues and tumor tissues. (B) The location of the ITGB1 gene is represented on chromosome 10 using the TCGA‐STAD cohort. (C) Pan‐cancer analysis showed the ITGB1 protein expression in tumor tissue. (D) Negative staining of ITGB1 in normal gastric mucosa. (E–G) Weak (E), moderate (F), and strong ITGB1 staining (G) in gastric cancer tissues (immunohistochemical staining, ×200).

In the pan‐cancer analysis, compared with normal tissue, ITGB1 is significantly overexpression in various tumors, including stomach adenocarcinoma (STAD), head and neck squamous cell carcinoma (HNSC), esophageal carcinoma (ESCA), cholangiocarcinoma (CHOL) pheochromocytoma and paraganglioma (PCPG), renal clear cell carcinoma (KIRC), and hepatocellular carcinoma (LIHC) (Figure 1A, *p* < 0.05). The genomic location of human ITGB1 is on chromosome 10 (Figure 1B). The data from the Human Protein Atlas indicated cytoplasmic and membranous staining in a variety of cancer cells (Figure 1C).

### 3.2 ITGB1 overexpression in gastric cancer and correlation with clinicopathological characteristics

**FIGURE 2:**
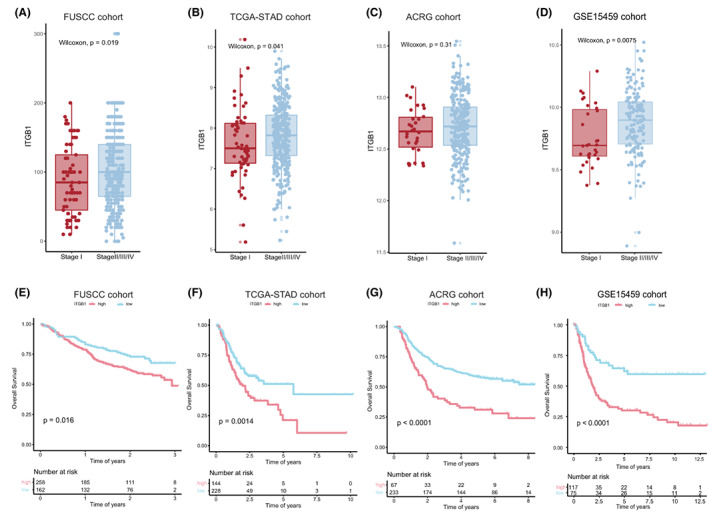
High ITGB1 expression correlates with a poor prognosis in advanced gastric cancer. (A–D) High ITGB1 expression was related to advanced gastric cancer in the FUSCC, TCGA‐STAD, ACRG, and GSE15459 cohorts. (E–H) ITGB1 is a prognostic factor that impacts of the overall survival of gastric cancer in the TCGA‐STAD, FUSCC, ACRG, and GSE15459 cohorts.

In the FUSCC cohort, ITGB1 was expressed in the cytoplasm of tumor cells. The positive rate of ITGB1 in GC tumor tissues was 61.4% (258/420), which was much higher than that in the normal gastric mucosa (33.3%, 9/27, Figure 1D–G). The relationship between ITGB1 protein expression and relevant clinicopathological features is summarized in Table 1. ITGB1 expression in GC was significantly correlated with patient's age (*p* < 0.001), tumor differentiation (*p* = 0.001), and Lauren's classification (*p* < 0.001). Positive ITGB1 protein expression was detected in 77.9% (148/190) of intestinal‐type cancers, 40.7% (59/145) of diffuse‐type cancers, 53.1% (34/64) of mixed‐type cancers, and 77.8% (14/18) of indeterminate‐type cancers. Positive ITGB1 staining was also associated with the depth of tumor invasion (*p* = 0.017) and clinical stage (*p* = 0.011). ITGB1 expression was observed in 42.9% (21/49) of T1‐stage cancers, 63.0% (34/54) of T2‐stage cancers, and 64.0% (203/317) of T3 + T4‐stage cancers (Figure 2A). The positive expression rate of ITGB1 was higher in advanced‐stage tumors than in those in the early stage. The same results were found in the TCGA‐STAD and GSE15459 cohort (Figure 2B, *p* = 0.041, Figure 2D, *p* = 0.0075, respectively). A trend of increasing ITGB1 expression was noted in advanced GC in the ACRG cohort (Figure 2C, *p* = 0.31). The relationships between ITGB1 overexpression and the clinicopathological characteristics of the main GC subtypes of Lauren's classification, the intestinal‐type GC, and the diffuse‐type GC, were also analyzed (Table S1). In the diffuse‐type GC, ITGB1 expression was correlated with advanced T stage (*p* = 0.046) and TNM stage (*p* = 0.021) but not in the intestinal‐type GC. There was no relationship found between ITGB1 expression and lymph node metastasis (Table 1, *p* = 0.863). The correlations between ITGB1 expression and the overall survival (OS) and (disease‐free survival) DFS were also analyzed in FUSCC samples. As shown in Figures 2E and Figure S1A, compared with the prognosis of patients with negative ITGB1 expression, the positive ITGB1 expression correlated with shortened OS and DFS in GC patients (Figure 2E, *p* = 0.016, Figure S1A, *p* = 0.014, respectively). In the cohort of intestinal‐type GC patients, the positive ITGB1 expression also negatively correlated with OS and DFS (Figure S2A, *p* = 0.01, Figure S2B, *p* = 0.014, respectively). And a trend of shortened OS and DFS was observed in the ITGB1 overexpression diffuse‐type GC patients (Figure S2C, *p* = 0.11, S2D, *p* = 0.075, respectively). The TCGA‐STAD, ACRG, and GSE15459 data sets also showed that patients with high ITGB1 expression had a significantly poor prognosis (Figure 2F–H, *p* < 0.01; Figure S1B, *p* < 0.01).

**TABLE 1:** Relationship between ITGB1 expression and clinicopathological features in gastric cancer (the FUSCC cohort)

Clinicopathological features	ITGB1+ (*n* = 258)	ITGB1‐ (*n* = 162)	*p* value
Gender			0.233
Male	184	106	
Female	74	56	
Age			<0.001
≥60 years	105	96	
<60 years	153	66	
Size			0.711
≥5 cm	97	58	
<5 cm	161	104	
Differentiation			0.001
High grade	180	136	
Low grade	78	26	
Histological subtype			<0.001
Intestinal	148	42	
Diffused	59	86	
Mixed	34	30	
Indeterminate	14	4	
Depth of invasion			0.017
T1	21	28	
T2	34	20	
T3 + T4	203	114	
Lymph node metastases			0.863
Negative	76	49	
Positive	182	113	
Clinical stage			0.011
I	33	36	
II/III/IV	225	126	

### 3.3 The ITGB1 overexpression correlates with activation of the Wnt/β‐catenin signaling pathway in gastric cancer

**FIGURE 3:**
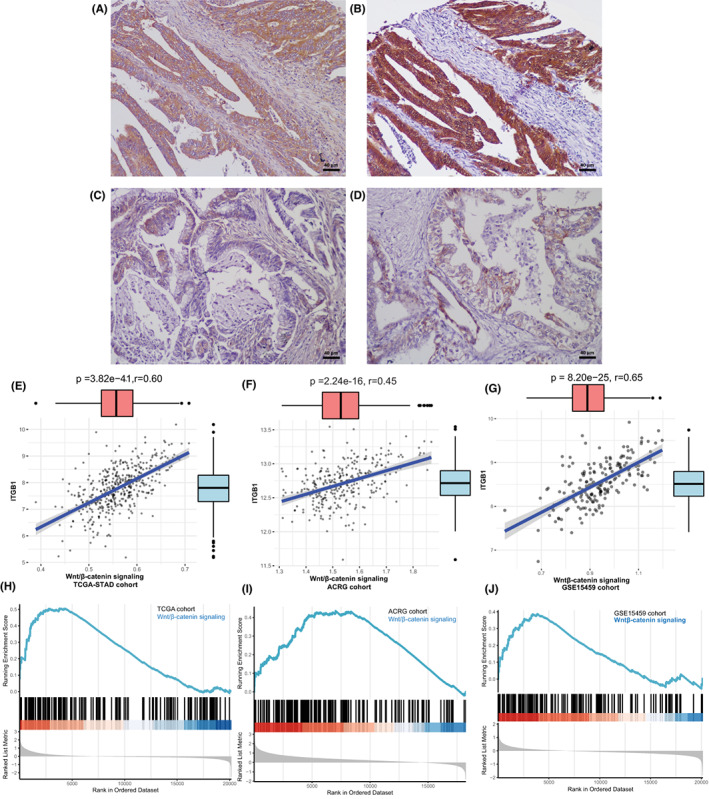
ITGB1 expression is correlated with activation of the Wnt/β‐catenin signaling pathway. (A–B) Coexpression of ITGB1 (A) and β‐catenin (B) in the nucleus/cytoplasm in some areas of gastric cancer (immunohistochemical staining, ×200). (C and D) Low ITGB1 expression (C) was related to β‐catenin expression (D) in the gastric cancer cell membrane (immunohistochemical staining, ×200). (E–G) A significant relationship between ITGB1 expression and the Wnt/β‐catenin signaling pathway in the TCGA‐STAD, ACRG, and GSE15459 cohorts. (H–J) GSEA plot showing that the Wnt/β‐catenin signaling pathway is activated in gastric cancer patients with high ITGB1 expression.

The Wnt/β‐catenin signaling pathway has been identified to be a core mediator of signaling downstream of the oncogenic functions of integrin family members.^9^, ^23^, ^24^ β‐catenin is a key molecule of the Wnt signaling pathway, and nuclear/cytoplasmic localization of β‐catenin can be used as a marker of Wnt signaling pathway activation. First, we observed the correlation of ITGB1 expression with β‐catenin localization in FUSCC samples. β‐catenin IHC staining was performed in 387 cases. In ITGB1‐positive staining cases (*n* = 248), nuclear/cytoplasmic accumulation of β‐catenin was detected in 69 cases (69/248, 27.8%). In ITGB1‐negative cases (*n* = 139), 15.1% cases (21/139) showed accumulation of β‐catenin in nucleus/cytoplasm (*p* = 0.005, Table 2). In ITGB1‐positive cases, accumulation of β‐catenin in nucleus/cytoplasm was observed in 33.9% diffuse‐type cases (20/59) and 25.5% intestinal‐type cases (36/141), while in 20.0% cases of ITGB1‐negative diffuse‐type GC (17/85) and 18.4% cases of ITGB1‐negative intestinal‐type GC (7/38) (*p* = 0.061, *p* = 0.362, respectively, Table 2). Compared with the ITGB1‐negative group, nuclear/cytoplasmic accumulation of β‐catenin was observed more frequently in the ITGB1‐positive staining group. Additionally, IHC staining showed that ITGB1 prominently colocalized with β‐catenin in some areas of tumor specimens (Figure 3A–D).

**TABLE 2:** Relationships between ITGB1 expression and localization of β‐catenin in gastric cancer (the FUSCC cohort)

β‐catenin	Total cases		*p* value	Intestinal type		*p* value	Diffuse type		*p* value
	ITGB1 ‐ (*n* = 139)	ITGB1 + (*n* = 248)		ITGB1 ‐ (n = 38)	ITGB1 + (*n* = 141)		ITGB1 ‐ (n = 85)	ITGB1 + (*n* = 59)	
Lost expression/membrane	118	179	0.005	31	105	0.362	68	39	0.061
Nucleus/cytoplasm	21	69		7	36		17	20	

To establish the relevance of ITGB1 and the Wnt/β‐catenin signaling pathway in human GC, we stratified patients in the TCGA‐STAD/ACRG/GSE15459 cohorts by ITGB1 expression and found a significantly high correlation between ITGB1 and the Wnt/β‐catenin signaling pathway (Figure 3E–G, *p* < 0.001). The GSEA results also showed that the Wnt/β‐catenin signaling pathway was activated in patients with high ITGB1 expression (Figure 3H–J, *p* < 0.01, NES = 1.294162, NES = 1.596521, NES = 1.752213). The results from the FUSCC, TCGA‐STAD, ACRG, and GSE15459 cohorts indicated that ITGB1 expression is correlated with activation of the Wnt/β‐catenin signaling pathway in GC.

### 3.4 ITGB1 overexpression correlates with immunosuppressive factors in gastric cancer

**FIGURE 4:**
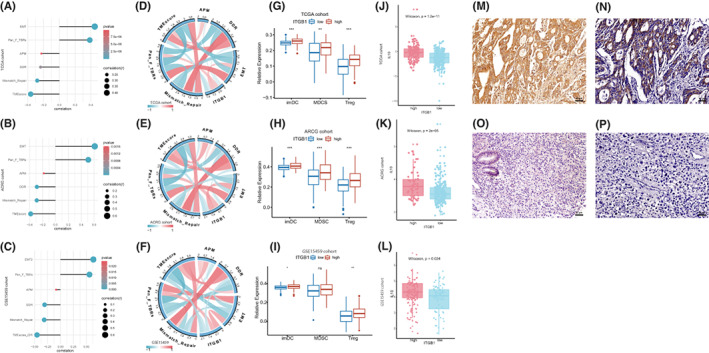
ITGB1 is associated with tumor microenvironment signatures. (A–F) ITGB1 was highly correlated with the tumor microenvironment signature in the TCGA‐STAD, ACRG, and GSE15459 cohorts. (G–I) imDCs, MDSCs, and Tregs were elevated in the TCGA‐STAD, ACRG, and GSE15459 cohorts with high ITGB1 expression. (J–L) Boxplot showing that IL‐10 was significantly correlated with ITGB1 expression in the TCGA‐STAD, ACRG, and GSE15459 cohorts. (M–P) ITGB1 (M) coexpression with IL‐10 (N) in gastric cancer cells (immunohistochemical staining, ×200). (O and P) Low ITGB1 (O) expression was associated with low IL‐10 (P) expression (immunohistochemical staining, ×200).

ITGB1 is one of the upstream molecules of the Wnt/β‐catenin signaling pathway, and we found that it is correlated with activation of the Wnt/β‐catenin signaling pathway in GC. The relationship between ITGB1 and tumor immunosuppression in GC needs to be explored. In the TCGA‐STAD/ACRG/GSE15459 cohorts, our analysis results showed a positive correlation between ITGB1 expression and epithelial–mesenchymal transition (EMT) and the panfibroblast TGF/beta response signature (Pan_F_TBRs) (Figure 4A–F, *p* < 0.001). High ITGB1 expression was negatively correlated with antigen processing machinery (APM), DNA damage repair (DDR), mismatch repair, and TMEscore signatures (Figure 4A–F, *p* < 0.001). Correlations between ITGB1 expression and representative immature dendritic cell (imDC), myeloid‐derived suppressor cell (MDSC), and regulatory T cell (Treg) signaling genes were found in the GC databases for the TCGA‐STAD and ACRG cohorts (Figure 4G–H, *p* < 0.01). The imDC and Treg were also highly expressed in high ITGB1 groups in GSE15459 cohort (Figure 4I). These results indicated that ITGB1 correlated with immunosuppressive factors in GC.

Immunosuppressive cells promote immune escape by producing immunosuppressive cytokines in the tumor microenvironment, resulting in dysfunctional T cells. Therefore, we stratified patients in the TCGA‐STAD/ACRG/GSE15459 cohorts by ITGB1 expression and analyzed the expression of immunosuppressive factors in these two subgroups. We found elevated expression of IL‐10 in the ITGB1 expression group (Figure 4J–L), which was verified by IHC in the FUSCC cohort. IL‐10 expression was detected in 52 GC tissue samples (Figure 4M–P). In the positive‐ITGB1 expression group, IL‐10 expression was detected in 80.6% (25/31) of tumor cells, whereas in the negative‐ITGB1 expression group, only 28.6% (6/21) of tumor cells showed IL‐10 expression (*p* < 0.001). Consistent with the results of the TCGA‐STAD/ACRG/GSE15459 cohorts, ITGB1‐positive GC showed a higher expression level of the immunosuppressive factor IL‐10. Moreover, we evaluate the immune checkpoint, such as PD‐L1, CTLA‐4, TIM3, IDO1, LAG3 PD‐1 to be confirmed the immunosupressive TME in GC which may relate to ITGB1 expression. The results showed that PD‐L1 and TIM3 were significantly highly expressed in high ITGB1 groups (Figure S3A–C, *p* < 0.05).

### 3.5 ITGB1 as a potential marker for predicting immunotherapy effects in gastric cancer

**FIGURE 5:**
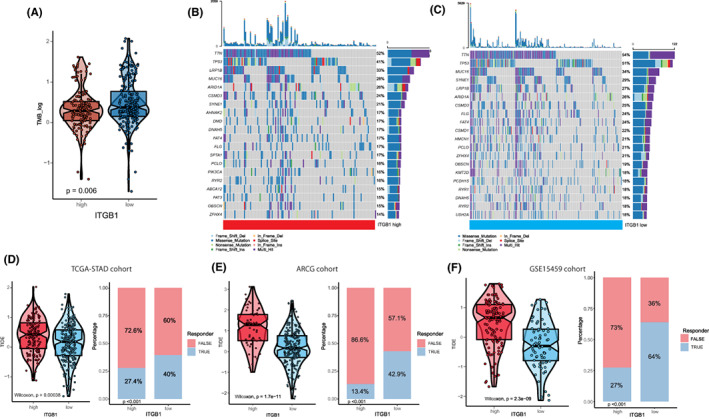
Correlation between ITGB1 and the tumor mutation burden of gastric cancer. ITGB1 may act as a potential predictor of the immunotherapy response in gastric cancer. (A) The significantly different distributions of the TMB in high‐ and low‐ITGB1 expression patients in the TCGA‐STAD cohort (*p* = 0.006). (B and C) The mutation landscape of the top 20 most frequently mutated genes in the TCGA‐STAD cohort. (D–F) Violin/box plot showing significantly different distributions of TIDE scores between high and low ITGB1 expression in the TCGA‐STAD, ACRG, and GSE15459 cohorts. The prediction of response rates of immunotherapies in patients with high and low ITGB1 expression in the TCGA‐STAD, ACRG, and GSE15459 cohorts.

We further explored the mutation landscapes of patients with high and low ITGB1 expression levels. We found that patients with low ITGB1 expression had a significantly high tumor mutation burden (Figure 5A, *p* = 0.006). The oncoplot indicated that the low‐ITGB1 subgroups presented more extensive mutations in the top 20 genes (Figure 5B–C). Next, TIDE was used to predict the immunotherapy response in the TCGA‐STAD/ACRG/GSE15459 cohorts. Notably, patients with low ITGB1 expression exhibited significantly low TIDE scores and significantly high response ratios (Figure 5D–F, *p* < 0.001), which may indicate that ITGB1 is a potential predictor of the efficacy of cancer immunotherapy in GC.

## 4 DISCUSSION

Our study indicated that ITGB1‐positive expression in GC was significantly associated with a higher T stage, an advanced clinical stage, and worse DFS and OS in our FUSCC cohort. By analyzing the available data from the TCGA‐STAD/ACRG/GSE15459 cohorts, we also found ITGB1 high expression in GC was related to a poor prognosis of GC patients. However, no significant difference was found between early stage and advanced‐stage GC with ITGB1 expression in the ACRG cohort, which could be as a consequence of the limited number of patients. Thus, our findings suggest that ITGB1 plays a critical role in the progression of GC and has the potential to be a prognostic predictor in GC.

Since the Wnt/β‐catenin signaling pathway involved in transducing the oncogenic signaling of ITGB1 in NSCLC,^9^, ^24^ the potential influence of ITGB1 on the Wnt/β‐catenin signaling pathway in GC was investigated. We found that expression of β‐catenin in nucleus and cytoplasm was upregulated in ITGB1‐positive group compared with the negative group in our own cohort. Consistently, we stratified patients in the TCGA‐STAD/ACRG/GSE15459 cohorts by ITGB1 expression and found a significant correlation between ITGB1 and upregulation of the Wnt/β‐catenin signaling pathway.

Cancer‐induced immune suppression is a major problem resulting in resistance to immunotherapies. However, the mechanisms of cancer‐induced immune suppression are still not fully understood. Increasing evidence has revealed that the oncogenic roles of the Wnt/β‐catenin signaling pathway are not only engaged in controlling cell proliferation, differentiation, and survival but are also associated with immune suppression in various cancers. Immunosuppressive cells, such as Tregs, regulatory dendritic cells (Dcregs), and MDSCs, are recruited by aberrant activation of the Wnt/β‐catenin signaling pathway in tumor microenvironment. Ji et al. showed that activation of β‐catenin in GC caused Treg recruitment and contributed to the progression of GC.^25^ In human melanoma, the activated Wnt/β‐catenin signaling pathway was reported to impair dendritic cell maturation and to induce Dcregs.^26^ For MDSCs, chemokine ligand 2 (CCL2) is a major recruiter of MDSCs. The expression of CCL2 was regulated by activation of β‐catenin. The β‐catenin/TCF‐4 complex directly bound to the CCL2 promoter to upregulate CCL2 expression in breast cancer^27^ and colorectal cancer.^28^ We found that the signaling genes related to immature dendritic cells, Tregs, and MDSCs were all positively correlated with ITGB1 expression in the TCGA‐STAD/ACRG/GSE15459 cohorts, indicating that GCs with higher ITGB1 expression usually exhibit more immunosuppressive microenvironment features.

Next, we found elevated expression of IL‐10 in the positive ITGB1 expression group in our own cohort and TCGA‐STAD/ACRG/GSE15459 data sets. IL‐10, as an immunosuppressive cytokine, can be produced by monocytes or even tumor cells. The expression of IL‐10 was also regulated by activation of β‐catenin in human melanoma^29^ and GC cell lines (data not shown). The IL‐10 promoter contained TCF binding motifs for regulation by the β‐catenin/TCF‐4 complex in human melanoma.^26^ We suggest that upregulated ITGB1 in GC may promote immune suppression by elevating immunosuppressive cytokines in the tumor microenvironment. The Wnt/β‐catenin signaling pathway may also play an important role in this process.

Previous studies indicated that the Wnt/β‐catenin signaling pathway is implicated in the process of EMT and TGF/beta signaling pathway‐related fibrosis.^30^, ^31^ EMT and the panfibroblast TGF/beta response signature (Pan_F_TBRs) activate the stroma to suppress immune activation due to T cell suppression.^32^, ^33^ In the TCGA‐STAD /ACRG/GSE15459 cohorts, we found that ITGB1 showed a positive correlation with EMT and Pan_F_TBR signatures. On the other hand, activation of DNA damage repair (DDR), antigen processing machinery (APM), mismatch repair, and the inflamed TME phenotype indicated the immune response activation of the tumor microenvironment.^34^, ^35^ Therefore, negative correlations between ITGB1 expression and DDR, APM, mismatch repair, and TMEscore signatures were found in the TCGA‐STAD/ACRG/GSE15459 cohorts, suggesting that high‐ITGB1 subgroups may be classified as immune‐suppressive subgroups.

The TMB has been considered a potential biomarker to predict immunotherapy effects. We found that ITGB1 expression was negatively related to an elevated TMB in the TCGA‐STAD cohort. Therefore, we used TIDE to predict the immunotherapy effect in the TCGA‐STAD and ACRG cohorts. The low‐ITGB1 subgroups were significantly associated with a better response to immunotherapy. Immunosuppressive factors may attenuate the efficacy of immune checkpoint inhibitors in the high‐ITGB1 subgroups. Taken together, ITGB1 may act as a potential biomarker for predicting GC patients who may benefit from immune checkpoint inhibitors.

However, there are still some limitations in our study. First, further studies are needed to investigate the detailed mechanisms by which ITGB1 participates in the regulation of immunosuppression in the Wnt/β‐catenin signaling pathway‐activated GC. Second, ITGB1 as a potential predictor of effective immunotherapy strategies should be validated in a real‐world data of GC patients treated with immune checkpoint inhibitors.

## 5 CONCLUSIONS

High ITGB1 expression in GC indicates a poor prognosis and worse response to immunotherapy through activation of the Wnt/β‐catenin signaling pathway. ITGB1‐positive expression is correlated with some immunosuppressive factors in GC environment. ITGB1 might be an immunotherapeutic target in Wnt/β‐catenin signaling pathway‐activated GC, which needs to be confirmed by further studies.

## AUTHORS' CONTRIBUTIONS

LW and WS designed the study and supervised the research process. WG, HS, and MZ performed the experiments, collected, and analyzed data. SM, CT, SN, and ZY generated the figures and tables. WG, LW, and YW wrote the original draft. All authors contributed to this research and approved the final version.

## FUNDING INFORMATION

The current study was supported by grants from the National Natural Science Foundation of China (81972249, 81802367, 82172702), the Natural Science Foundation of Shanghai (18ZR1408000, 21ZR1414900), and the Hospital Foundation of Fudan University Shanghai Cancer Center (YJMS202002).

## CONFLICT OF INTEREST

The authors have no conflicts of interest to declare.

## ETHICS APPROVAL AND CONSENT TO PARTICIPATE

The use of clinical specimens and pathological data in this study was approved by The Clinical Research Ethics Committee of Fudan University Shanghai Cancer Center (FUSCC), and informed consent was obtained from all patients.

## Supporting information


Figures S1‐S3

Table S1

## Data Availability

The data sets generated and/or analyzed during the current study are available from the corresponding author on reasonable request.
