# Supplementary figures and images for: ITGB1 as a prognostic biomarker correlated with immune suppression in gastric cancer
Source: Cancer Med. 2022 Jul 21;12(2):1520–31. doi: 10.1002/cam4.5042 (PMC9883581; doi:10.1002/cam4.5042)

# Supplementary Figure 1

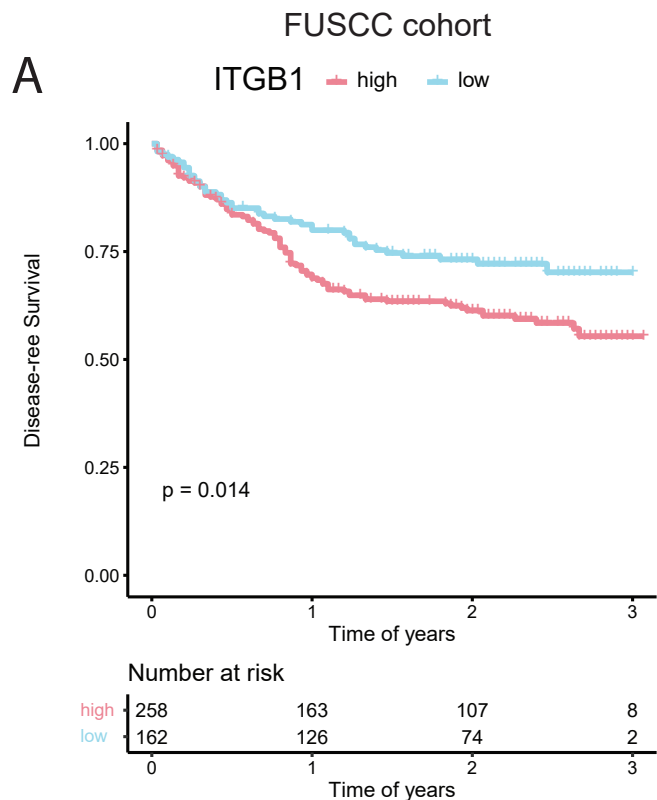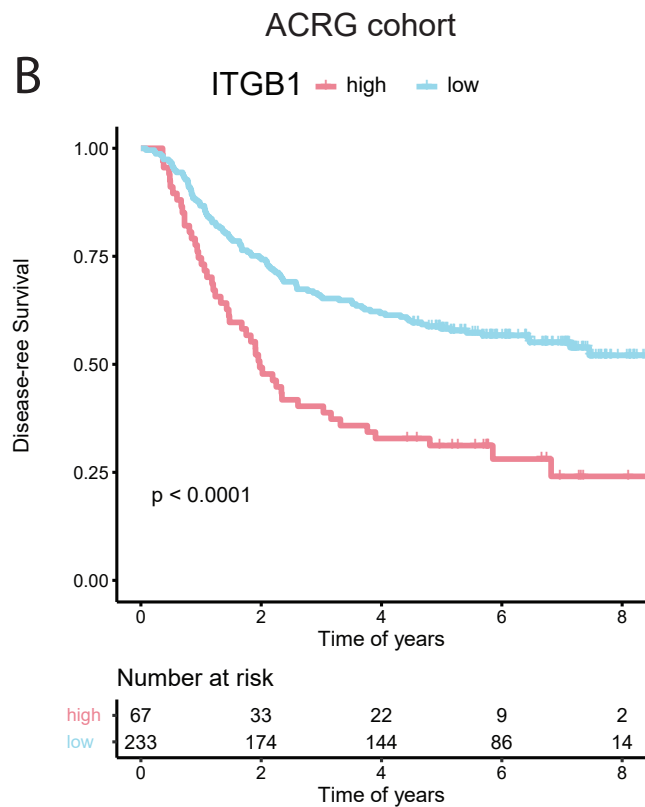

# Supplementary Figure 2

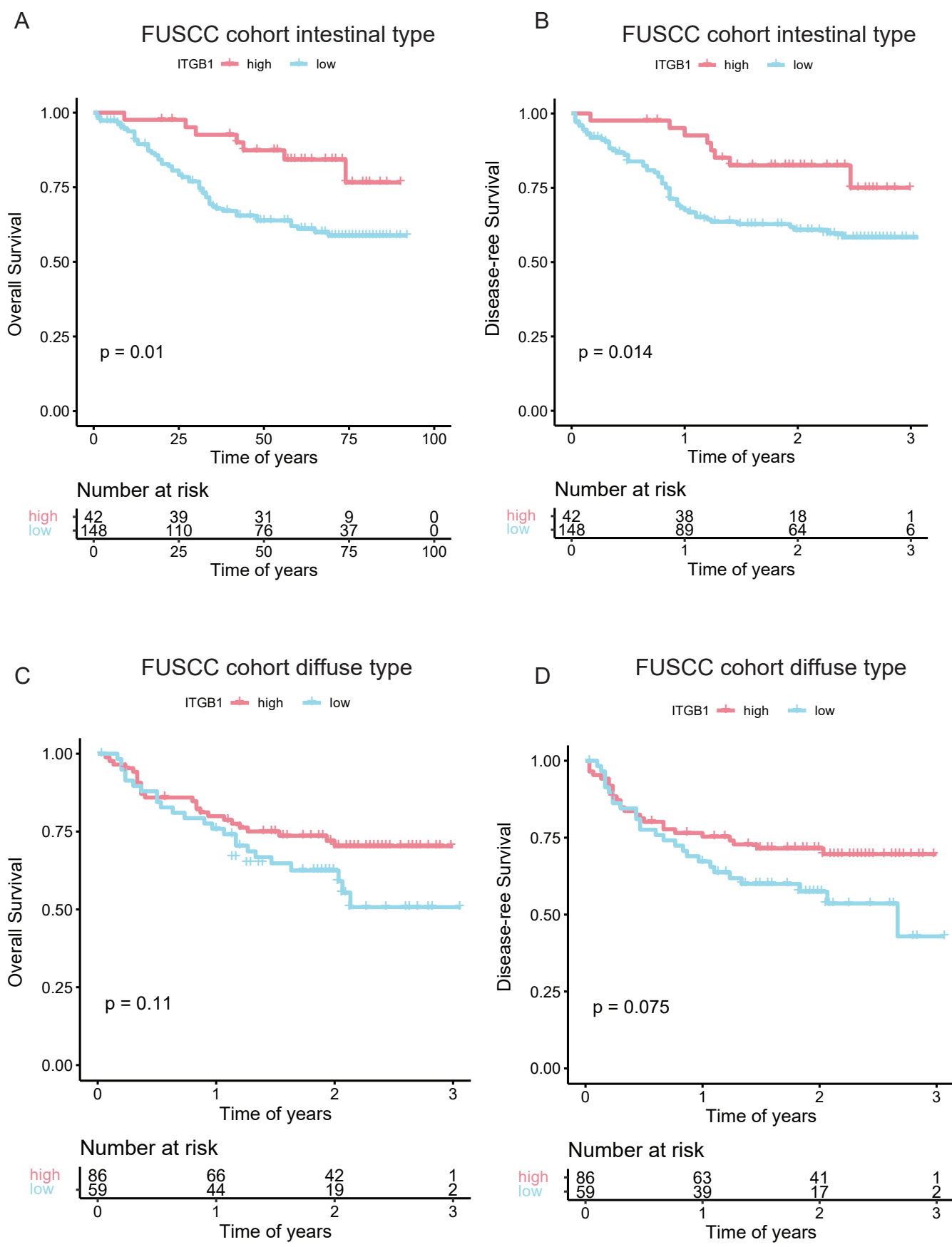

Supplementary Figure 3

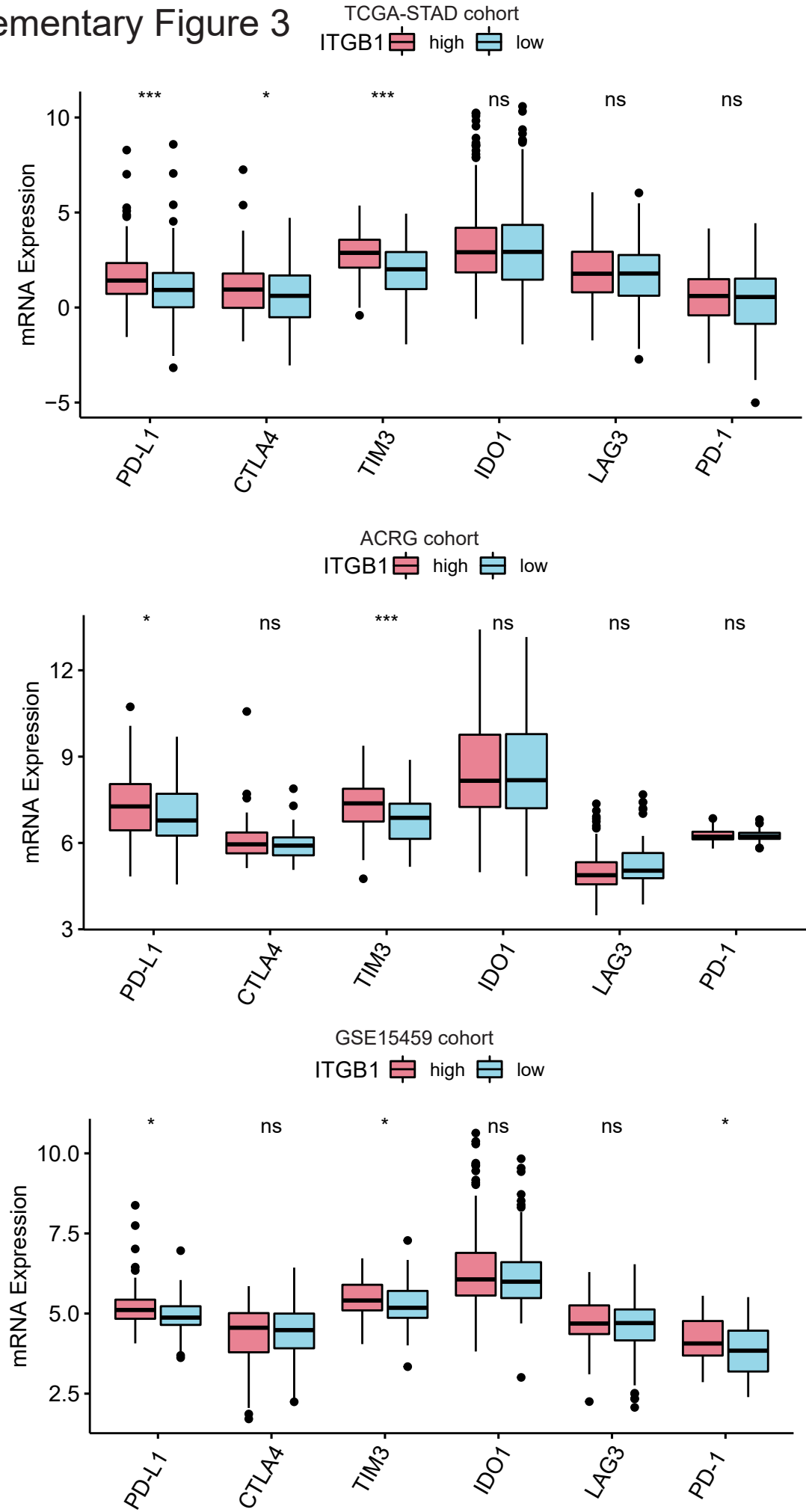

Supplement: Supplementary file 1 — Figures S1‐S3 [file CAM4-12-1520-s001.pdf]
