# Supplementary material for: ITGB1 as a prognostic biomarker correlated with immune suppression in gastric cancer
Source: Cancer Med. 2022 Jul 21;12(2):1520–31. doi: 10.1002/cam4.5042 (PMC9883581; doi:10.1002/cam4.5042)
Supplement: Supplementary file 2 — Table S1 [file CAM4-12-1520-s002.docx]

| Table S1. Relationships between ITGB1 expression and the clinicopathological features of the main  GC subtypes of Lauren’s classification   \| Clinicopathological features \| Intestinal type \| \| *P* value \| Diffuse type \| \| *P* value \| \| --- \| --- \| --- \| --- \| --- \| --- \| --- \| \| ITGB1+ (n=148) \| ITGB1- (n=42) \| ITGB1+ (n=59) \| ITGB1- (n=86) \| \| Gender \|  \|  \| 0.455 \|  \|  \| 0.785 \| \| Male \| 114 \| 30 \|  \| 37 \| 52 \|  \| \| Female \| 34 \| 12 \|  \| 22 \| 34 \|  \| \| Age \|  \|  \| 0.181 \|  \|  \| 0.228 \| \| ≥ 60 years \| 101 \| 24 \|  \| 25 \| 28 \|  \| \| < 60 years \| 47 \| 18 \|  \| 34 \| 58 \|  \| \| Size \|  \|  \| 0.127 \|  \|  \| 0.680 \| \| ≥ 5 cm \| 50 \| 9 \|  \| 22 \| 35 \|  \| \| < 5 cm \| 98 \| 33 \|  \| 37 \| 51 \|  \| \| Depth of invasion \|  \|  \| 0.261 \|  \|  \| 0.046 \| \| T1 \| 12 \| 7 \|  \| 6 \| 19 \|  \| \| T2 \| 25 \| 6 \|  \| 3 \| 10 \|  \| \| T3+T4 \| 111 \| 29 \|  \| 50 \| 57 \|  \| \| Lymph nodemetastases \|  \|  \| 0.232 \|  \|  \| 0.983 \| \| Negative \| 52 \| 19 \|  \| 15 \| 22 \|  \| \| Positive \| 96 \| 23 \|  \| 44 \| 64 \|  \| \| Clinical stage \|  \|  \| 0.431 \|  \|  \| 0.021 \| \| I \| 24 \| 9 \|  \| 7 \| 24 \|  \| \| II /III/IV \| 124 \| 33 \|  \| 52 \| 62 \|  \| |
| --- | --- | --- | --- | --- | --- | --- | --- | --- | --- | --- | --- | --- | --- | --- | --- | --- | --- | --- | --- | --- | --- | --- | --- | --- | --- | --- | --- | --- | --- | --- | --- | --- | --- | --- | --- | --- | --- | --- | --- | --- | --- | --- | --- | --- | --- | --- | --- | --- | --- | --- | --- | --- | --- | --- | --- | --- | --- | --- | --- | --- | --- | --- | --- | --- | --- | --- | --- | --- | --- | --- | --- | --- | --- | --- | --- | --- | --- | --- | --- | --- | --- | --- | --- | --- | --- | --- | --- | --- | --- | --- | --- | --- | --- | --- | --- | --- | --- | --- | --- | --- | --- | --- | --- | --- | --- | --- | --- | --- | --- | --- | --- | --- | --- | --- | --- | --- | --- | --- | --- | --- | --- | --- | --- | --- | --- | --- | --- | --- | --- | --- | --- | --- | --- | --- | --- | --- | --- | --- | --- | --- | --- | --- | --- | --- |
